# Enhanced surveillance for the detection of psoriatic arthritis in a UK primary care psoriasis population: results from the TUDOR trial

**DOI:** 10.1093/rheumatology/keae374

**Published:** 2024-07-22

**Authors:** Neil McHugh, William Tillett, Philip Helliwell, Jonathan Packham, Howard Collier, Claire Davies, Myka Ransom, Laura Coates, Sarah T Brown

**Affiliations:** Department of Life Sciences, University of Bath, Bath, UK; Royal National Hospital for Rheumatic Diseases, Royal United Hospitals Bath NHS Foundation Trust, Bath, UK; Department of Life Sciences, University of Bath, Bath, UK; Royal National Hospital for Rheumatic Diseases, Royal United Hospitals Bath NHS Foundation Trust, Bath, UK; Leeds Institute of Rheumatic and Musculoskeletal Medicine, University of Leeds, Leeds, UK; Rheumatology Department, Midlands Partnership NHS Foundation Trust, Haywood Hospital, Stoke on Trent, UK; Clinical Trials Research Unit, Leeds Institute of Clinical Trials Research, University of Leeds, Leeds, UK; Clinical Trials Research Unit, Leeds Institute of Clinical Trials Research, University of Leeds, Leeds, UK; Clinical Trials Research Unit, Leeds Institute of Clinical Trials Research, University of Leeds, Leeds, UK; Nuffield Department of Orthopaedics, Rheumatology and Musculoskeletal Sciences, University of Oxford, Botnar Research Centre, Oxford, UK; Clinical Trials Research Unit, Leeds Institute of Clinical Trials Research, University of Leeds, Leeds, UK

**Keywords:** psoriasis, psoriatic arthritis, screening, randomized control trial

## Abstract

**Objectives:**

Our objective was to determine whether early detection of undiagnosed PsA in a primary care psoriasis population improves outcome in physical function at 24 months post-registration.

**Methods:**

A multicentre, prospective, parallel group cluster randomized controlled trial in patients with psoriasis was conducted. Participants with suspected inflammatory arthritis on screening were referred for an assessment of PsA [enhanced surveillance (ES) arm: at baseline, and 12 and 24 months; standard care (SC) arm: at 24 months]. The primary outcome measure was the HAQ Disability Index (HAQ-DI) at 24 months post-registration in participants diagnosed with PsA.

**Results:**

A total of 2225 participants across 135 general practitioner practices registered: 1123 allocated to ES and 1102 to SC. The primary analysis population consisted of 87 participants with a positive diagnosis of PsA: 64 in ES, 23 in SC. The adjusted odds ratio (OR) for achieving a HAQ-DI score of 0 at 24 months post-registration in ES compared with SC was 0.64 [95% CI (0.17, 2.38)], and the adjusted OR of achieving a higher (non-zero) HAQ-DI score at 24 months post-registration in ES relative to SC arm was 1.12 (95% CI 0.67, 1.86), indicating no evidence of a difference between the two treatment groups (*P* = 0.66).

**Conclusion:**

The trial was underpowered for demonstrating the prespecified treatment effect; in patients with psoriasis there was no evidence that early diagnosis of PsA by ES in primary care changes physical function at 24 months compared with SC.

**Clinical trial registration:**

The TUDOR trial is registered as ISRCTN38877516.

Rheumatology key messages

- The benefit of early detection of PsA in a primary care psoriasis population remains unproven.
- Surveillance for undiagnosed PsA generally detects mild cases as measured by physical function.
- Findings from the TUDOR (Total bUrDen Of psoRiasis) trial raise questions about the value of screening everyone with psoriasis for PsA in a primary care setting.

## Introduction

Psoriasis is a common skin disorder that leads to the development of a chronic inflammatory arthritis termed PsA in about 20–30% of cases [1, 2]. People with PsA may have peripheral joint disease, axial disease and commonly other manifestations such as enthesitis, dactylitis and nail disease, that together compromise physical function, cause work disability and accrue significant health costs [3, 4]. Psoriasis precedes the development of PsA in most cases [5]. Therefore, the presence of psoriasis affords an excellent opportunity for the introduction of screening strategies that may detect PsA at an earlier stage than it would be in current practice.

Previous observational studies have suggested that delay in diagnosis of PsA is associated with poor outcome [6–8]. Patients with disease duration >2 years at referral sustain more joint damage than those with disease duration <2 years [6]. In a UK study a delay in diagnosis was an independent predictor of worse physical function after 10 years duration [7], findings confirmed in a Dublin cohort where even a 6-month delay contributed to peripheral joint erosion and worse long-term physical function [8]. In a prospective study from the Swedish Early Psoriatic Arthritis (SwePsA) Registry, a shorter delay between onset of symptom and diagnosis was an important predictor of favourable outcome at 5 years [9]. Hence, healthcare policy leaders such as the UK National Institute for Health and Care Excellence (NICE) recommend early referral in suspected cases, and that people with psoriasis receiving treatment are offered an annual assessment for PsA [10, 11].

Screening people with psoriasis for PsA in primary care and dermatology clinics has revealed a prevalence of undiagnosed disease in up to 29% of cases [12, 13]. However, the benefit from early detection of undiagnosed disease has never been investigated in a prospective randomized controlled trial. Here we report the findings at 24 months follow-up of participants enrolled in the Total bUrDen Of psoRiasis (TUDOR) trial that compared the clinical effectiveness of an enhanced surveillance (ES) strategy for the detection of PsA *vs* standard care (SC) in psoriasis patients recruited from primary care.

The primary objective of the trial was to determine whether the early detection of undiagnosed PsA in people with psoriasis by an ES intervention compared with SC improves outcome in physical function at 24 months. Secondary objectives were to compare disease activity and impact of disease between people diagnosed with PsA in the ES and SC arms at 24 months.

## Methods

### Study design

TUDOR is a multicentre, prospective, two-arm, parallel group cluster randomized controlled trial. The trial was conducted across a combination of primary care practices, for recruitment of patients with psoriasis but with no prior diagnosis of PsA, and at secondary care rheumatology clinics in the UK.

The trial had National Health Service (NHS) Health Research Authority Ethics approval (Reference: 16/SW/0161).

### Participants

Patients were eligible to participate in the trial if they were 18–70 years of age at the time of recruitment, had a diagnosis of psoriasis and no prior diagnosis of PsA, RA or axial SpA (axSpA).

Participants were identified at general practitioner (GP) practices (performing as Participant Identification Centres) by performing electronic searches of primary care records using pre-specified Read Codes list to include those who had a diagnosis of psoriasis but not a diagnosis of PsA, RA or axSpA. Potentially suitable participants were then screened by a GP within each of the GP practices to exclude patients thought to be inappropriate to approach. Potential participants were contacted via post (using a secure print company, Docmail), and sent an invitation pack which included an invitation letter, and a screening and demographic (including gender: male/female) questionnaire which was used to assess their eligibility including information on a recent diagnosis of PsA, axSpA or RA that may not have been included in their GP records. Participants choosing to consent (written informed consent) returned the completed screening and demographic questionnaire to Leeds Clinical Trials Research Unit (CTRU), who then conducted an eligibility check.

Eligible participants were registered using a 24-h automated web-based/telephone registration system based at Leeds CTRU. Participants registered onto the trial were managed according to either ES or SC depending on their GP practice.

### Randomization and masking

GP id="311" practices were randomized in a 1:1 allocation ratio to ES or SC. Randomization was performed using stratified random permuted blocks (block size 2), with stratification for GP practice list size (levels defined by the median list size: ≤7000/>7000 patients) and Central Commissioning Group (CCG); the randomization sequence was generated by the trial statistician at CTRU. Randomization of GP practices was conducted by Data Management staff based at the CTRU using a central and independent 24-h automated service provided by Leeds CTRU, thereby ensuring allocation concealment. Information on the allocation was provided to the GP (deputy) lead or a member of staff with a research related role at each GP practice.

All GP practice leads participating in the study were aware of the aims of the trial. Within the ES arm practices, only permanent GPs were informed of the trial objectives and design. Within the SC arm practices, to reduce the risk of any changes to the referral patterns occurring within those practices, steps were taken to minimize the number of GPs within a practice who were made aware of the trial objectives and design. To prevent raising participants’ awareness of musculoskeletal symptoms which may have led to a change in their behaviours, participants were informed that the study concerned the impact of psoriasis (all types and severity) on daily living and quality of life.

Due to the different frequency of assessments across both arms, ‘assessing clinicians’ at the secondary care rheumatology clinics were aware of treatment group allocation but were blinded to all questionnaire responses made by the participants. To reduce the risk of the treating clinician being aware of the participants’ involvement in the trial, GPs were asked to refer participants with positive signs and symptoms of inflammatory arthritis to secondary care for an assessment using usual referral procedures.

#### Procedures

*Enhanced surveillance (ES) arm:* at baseline, and 12 and 24 months post-registration participants underwent a clinical assessment by a research clinician for the presence of symptoms and signs of inflammatory arthritis, and completed questionnaires on psoriasis, health-related quality of life and physical functioning. Participants with suspected inflammatory arthritis were referred, via their GP, to the local rheumatology outpatient clinic.

*Standard care (SC) arm:* at baseline, and 12 and 24 months post-registration, participants completed postal questionnaires including on psoriasis, health-related quality of life and physical functioning. At 24 months, participants also underwent a clinical assessment by the research clinician as described above. Again, participants suspected with inflammatory arthritis were referred, via their GP, for a further assessment.

Participants in either arm of the study referred to rheumatology services as a result of routine clinical care by their GP at any time during follow-up were identified via completion of medical history questionnaires at each postal or annual assessment.

Clinical judgement formed the basis of the final PsA diagnosis though data sufficient to evaluate the CASPAR (ClASsification for Psoriatic ARthritis) criteria were also recorded. Participants diagnosed with PsA via a clinical diagnosis were invited to consent to complete PsA outcome questionnaires and a further clinical assessment at each standard clinic visit.

#### Outcomes

The primary outcome measure was participant reported physical function at 24 months post-registration in participants diagnosed with PsA, as measured by the HAQ Disability Index (HAQ-DI) [14], with a higher score representing greater disability.

The secondary outcome measures, PASDAS, a composite score of PsA disease activity [15], and PsAID-12, a self-reported questionnaire to assess the impact of PsA [16], were assessed in participants with a positive diagnosis of PsA, at routine standard care follow-up assessments. Adverse events or serious adverse events classified as related to the trial procedures were reported.

### Statistical analysis

The sample size was calculated using the primary endpoint, overall HAQ-DI score at 24 months post-registration. A total of 148 participants with PsA (74 in each arm) were required to be diagnosed with PsA to have 80% power for detecting the minimum clinically important difference (MCID) in the overall mean HAQ-DI scores of 0.35 units [17] at 24 months post-registration, assuming a two-sided *t*-test at the 5% significance level to be tested against the null hypothesis of the overall mean HAQ-DI values being the same in both groups. Under the pre-specified assumption of 18% [12, 18] of participants being diagnosed with PsA during the trial, 824 participants (412 per arm) were required for an individually randomized trial. This sample size was adjusted using an inflation factor to account for: mean and s.d. of 16.9 and 13.61 participants recruited per GP practice respectively, corresponding coefficient of variation of 0.81; an intra-cluster correlation coefficient of 0.03 [19–22] and attrition at the participant and GP practice level, assuming 40% and 3%, respectively.

A final target sample size of 2226 participants recruited across 133 GP practices ensured a minimum power of 80% for detecting the pre-specified MCID of 0.35 in the HAQ-DI.

The primary analysis of the primary endpoint was analysed on all participants with a diagnosis of PsA by allocated treatment group, with a sensitivity analysis on the complete case population, which included participants with complete outcome and covariate data.

The primary endpoint was analysed using a two-part zero-inflated beta regression model [23] to account for the tendency for HAQ-DI data to be skewed towards zero. HAQ-DI values were scaled by 3 due to the beta distribution being specified in the interval (0, 1). GP practice size, secondary care site and the baseline covariates age, scaled HAQ-DI score, socioeconomic status using the Index of Multiple Deprivation (IMD) [24] and treatment group were fitted in the model. As the logit link function is used, the model output is interpreted in terms of the odds of achieving a score of zero (zero inflated part) and the odds of achieving a higher HAQ-DI score (non-zero part). Continuous baseline covariates were centered around the mean value.

Missing covariate and outcome data were imputed prior to analysis using a multiple imputation model (50 imputed data sets generated) with the same covariates fitted as for the primary endpoint analysis model via predictive mean matching [25]. Adjusted parameter estimates were then combined using Rubin’s rules [26, 27].

The secondary outcomes, PASDAS and PsAID-12 for the participants diagnosed with PsA and who provided consent to additional questionnaires, are summarized descriptively over time from date of PsA diagnosis.

SAS version 9.4 was used for the statistical analysis.

The TUDOR trial is registered as ISRCTN38877516.

## Results

A total of 677 GP practices were invited to participate; of these, 135 GP practices were randomized (19.9%): 72 (53.3%) to ES, 63 (46.7%) to SC. A total of 28202 patients with a read code for psoriasis were identified; of these, 2225 were consented and recruited (7.9%): 1123 to ES, 1102 to SC. Of the 25 977 patients not recruited (92.1%), 4453 (17.1%) were deemed unsuitable to approach by the GP, 296 (1.1%) were ineligible and 21 109 (81.3%) did not consent; the study invitation letter was not sent out for the remaining 119 participants (0.5%).

**Figure 1.**
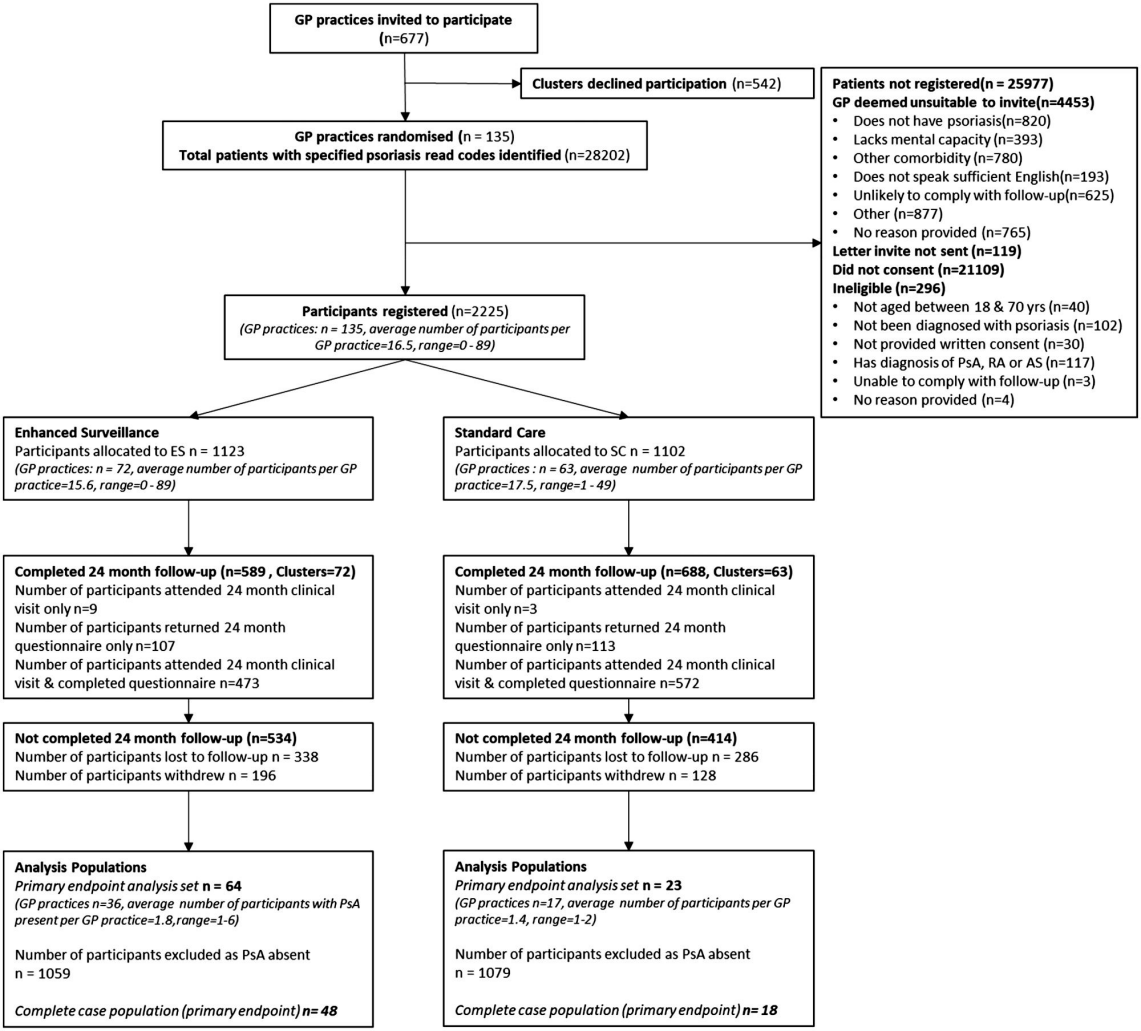
CONSORT (Consolidated Standards of Reporting Trials) flow diagram. GP: general practitioner; ES: enhanced surveillance; SC: standard care

The mean recruitment per GP practice was 16.5 participants (range 0–89); three (2.2%) GP practices opened but no participants were registered. A total of 324 (14.6%) participants withdrew and 624 (28.0%) were lost to follow-up prior to the 24-month assessment. The primary analysis population consisted of 87 participants with a positive diagnosis of PsA: 64 in ES, 23 in SC. Of these, 64 (73.6%) fulfilled CASPAR classification criteria for PsA, 15 (17.2%) did not and for 8 others (9.2%) data was not known or missing [28]. The CONSORT (Consolidated Standards of Reporting Trials) flow diagram in shown in Fig. 1.

Participants were recruited between 11 November 2016 and 22 June 2018. The last participant last visit was on 26 July 2021.

Tables 1 and 2 summarize the demographic and baseline characteristics for all participants recruited and for participants with a positive diagnosis of PsA respectively. The mean age of all participants recruited was 51 years (range 18, 71), 54.4% were female and almost all (96.1%) were white ethnicity. The mean (s.d.) BMI was 27.6 kg/m^2^ (16.5), median [interquartile range (IQR)] age at psoriasis diagnosis was 25 (15, 40) and the median (IQR) baseline HAQ score was 0.0 (0.00, 0.13). The baseline characteristics were similar across both treatment groups.

**Table 1.** Baseline characteristics for all registered participants

	Enhanced surveillance (*N* = 1123)	Standard care (*N* = 1102)	Total (*N* = 2225)
Age, years			
Mean (s.d.)	51.1 (13.35)	51.3 (13.24)	51.2 (13.24)
Median (range)	53.2 (18.5, 71.0)	52.6 (18.1, 71.0)	53.0 (18.1, 71.0)
IQR	40.6, 62.5	41.9, 62.5	41.4, 62.5
Missing	0	0	0
Gender, *n* (%)			
Male	512 (45.6)	499 (45.3)	1011 (45.4)
Female	609 (54.2)	601 (54.5)	1210 (54.4)
Missing	2 (0.2)	2 (0.2)	4 (0.2)
Ethnicity, *n* (%)			
White	1079 (96.1)	1060 (96.2)	2139 (96.1)
Asian/Asian British	22 (2.0)	20 (1.8)	42 (1.9)
Mixed/Multiple ethnic groups	8 (0.7)	10 (0.9)	18 (0.8)
Black/African/Caribbean/Black British	2 (0.2)	1 (0.1)	3 (0.1)
Other ethnic group	2 (0.2)	3 (0.3)	5 (0.2)
Not stated	1 (0.1)	3 (0.3)	4 (0.2)
Missing	9 (0.8)	5 (0.5)	14 (0.6)
BMI (kg/m^2^)			
Mean (s.d.)	27.7 (5.94)	27.4 (5.63)	27.6 (5.79)
Median (range)	26.8 (15.0, 65.1)	26.6 (14.9, 89.2)	26.6 (14.9, 89.2)
IQR	23.6, 30.4	23.8, 30.0	23.7, 30.2
Missing	61	69	130
Age at psoriasis diagnosis			
Mean (s.d.)	27.3 (16.56)	27.9 (16.46)	27.6 (16.51)
Median (range)	25.0 (1.0, 69.0)	24.5 (0.0, 70.0)	25.0 (0.0, 70.0)
IQR	14.0, 40.0	15.0, 40.0	15.0, 40.0
Missing	63	56	119
Participant socioeconomic status (IMD)			
Mean (s.d.)	6.7 (2.67)	6.9 (2.73)	6.8 (2.70)
Median (range)	7.0 (1.0, 10.0)	7.0 (1.0, 10.0)	7.0 (1.0, 10.0)
IQR	5.0, 9.0	5.0, 9.0	5.0, 9.0
Missing	14	13	27
Secondary care centre, *n* (%)			
West Yorkshire—St Luke’s Hospital & Chapel Allerton Hospital	336 (29.9)	439 (39.8)	775 (34.8)
Stoke on Trent—Haywood Hospital	310 (27.6)	328 (29.8)	638 (28.7)
Bath—Royal National Hos. for Rheumatic Disease	477 (42.5)	335 (30.4)	812 (36.5)
GP practice list size, *n* (%)			
Below the median (≤7000)	186 (16.6)	150 (13.6)	336 (15.1)
Above the median (>7000)	937 (83.4)	952 (86.4)	1889 (84.9)
Total HAQ-DI score			
Mean (s.d.)	0.18 (0.42)	0.15 (0.40)	0.16 (0.41)
Median (Range)	0.00 (0.00, 3.00)	0.00 (0.00, 2.63)	0.00 (0.00, 3.00)
IQR	0.00, 0.13	0.00, 0.00	0.00, 0.13
Number with completed data, *N*	830	958	1788
Number of patients with insufficient data^a^	2	5	7
Number of patients missing baseline questionnaire	291	139	430

a Total HAQ-DI score is not computed when answers to fewer than six categories are provided. IMD: Index of Multiple Deprivation; IQR: interquartile range.

**Table 2.** Baseline characteristics for participants diagnosed with PsA by treatment group

	Enhanced surveillance (*N* = 64)	Standard care (*N* = 23)	Total (*N* = 87)
Age, years			
Mean (s.d.)	53.4 (11.81)	51.7 (11.63)	52.9 (11.71)
Median (range)	55.5 (29.0, 70.6)	49.6 (31.9, 70.3)	54.9 (29.0, 70.6)
IQR	42.5, 63.5	44.3, 61.6	42.7, 63.1
Missing	0	0	0
Gender, *n* (%)			
Male	34 (53)	11 (48)	45 (51.7)
Female	30 (47)	12 (52)	42 (48.3)
Ethnicity, *n* (%)			
White	56 (88)	19 (83)	75 (86.2)
Missing	8 (13)	4 (17)	12 (13.8)
BMI (kg/m^2^)			
Mean (s.d.)	27.5 (6.93)	29.3 (4.68)	27.9 (6.45)
Median (range)	26.3 (18.6, 64.5)	28.0 (23.4, 40.7)	26.4 (18.6, 64.5)
IQR	24.0, 29.3	25.4, 32.5	24.4, 30.1
Missing	11	5	16
Age at psoriasis diagnosis			
Mean (s.d.)	26.9 (16.30)	25.4 (14.39)	26.5 (15.73)
Median (range)	24.0 (5.0, 65.0)	23.0 (10.0, 55.0)	23.5 (5.0, 65.0)
IQR	14.0, 36.0	12.0, 37.0	14.0, 36.5
Missing	11	4	15
Participant socioeconomic status (IMD)			
Mean (s.d.)	6.3 (2.68)	6.6 (3.00)	6.4 (2.75)
Median (range)	6.5 (1.0, 10.0)	6.0 (1.0, 10.0)	6.0 (1.0, 10.0)
IQR	4.0, 9.0	4.0, 10.0	4.0, 9.0
Missing	0	0	0
Secondary care centre, *n* (%)			
West Yorkshire—St Luke’s Hospital & Chapel Allerton Hospital	12 (19)	7 (30)	19 (21.8)
Stoke on Trent—Haywood Hospital	18 (28)	7 (30)	25 (28.7)
Bath—Royal National Hos. for Rheumatic Disease	34 (53)	9 (39)	43 (49.4)
GP practice list size, *n* (%)			
Below the median (≤7000)	11 (17)	4 (17)	15 (17.2)
Above the median (>7000)	53 (83)	19 (83)	72 (82.8)

IMD: Index of Multiple Deprivation; IQR: interquartile range.

The primary analysis population consisted of 87 participants with a positive diagnosis of PsA: 64 in ES, 23 in SC. The complete case population for the primary endpoint analysis consisted of 66 participants: 48 in ES, 18 in SC.

Tables 3 and 4 show the summary statistics for the primary outcome, overall HAQ-DI score, unscaled and scaled respectively in the primary analysis population. Supplementary Fig. S1, available at *Rheumatology* online presents histograms of the scaled overall HAQ-DI score (0–1) at baseline, and 12 and 24 months post-registration for the primary analysis population by treatment group. Supplementary Table S1, available at *Rheumatology* online summarizes the referral assessment time point for participants diagnosed with PsA.

**Table 3.** Overall unscaled HAQ-DI score (0–3) at baseline, and 12 and 24 months post-registration

	Enhanced surveillance (*N* = 64)	Standard care (*N* = 23)	Total (*N* = 87)
Baseline HAQ-DI scores			
All participants with a positive diagnosis of PsA			
Mean (s.d.)	0.28 (0.45)	0.34 (0.52)	0.29 (0.46)
Median (range)	0.00 (0.00, 2.13)	0.13 (0.00, 2.13)	0.00 (0.00, 2.13)
IQR	0.00, 0.38	0.00, 0.50	0.00, 0.38
0, *n* (%)	34 (53.1)	9 (39.1)	43 (49.4)
>0, *n* (%)	30 (46.9)	11 (47.8)	41 (47.1)
Missing, *n* (%)	0 (0.0)	3 (13.0)	3 (3.4)
Participants with non-zero score			
Mean (s.d.)	0.59 (0.49)	0.61 (0.58)	0.60 (0.51)
Median (range)	0.44 (0.13, 2.13)	0.38 (0.13, 2.13)	0.38 (0.13, 2.13)
IQR	0.25, 0.88	0.25, 0.75	0.25, 0.75
12-month HAQ-DI scores			
Mean (s.d.)	0.39 (0.45)	0.55 (0.67)	0.43 (0.52)
Median (range)	0.25 (0.00, 1.88)	0.25 (0.00, 2.00)	0.25 (0.00, 2.00)
IQR	0.00, 0.63	0.00, 1.00	0.00, 0.69
0, *n* (%)	18 (28.1)	7 (30.4)	25 (28.7)
>0, *n* (%)	35 (54.7)	12 (52.2)	47 (54.0)
Missing , *n* (%)	11 (17.2)	4 (17.4)	15 (17.2)
Participants with non-zero score			
Mean (s.d.)	0.59 (0.44)	0.88 (0.65)	0.66 (0.51)
Median (range)	0.50 (0.13, 1.88)	0.75 (0.13, 2.00)	0.50 (0.13, 2.00)
IQR	0.25, 0.88	0.25, 1.44	0.25, 0.88
24-month HAQ-DI scores			
All participants with a positive diagnosis of PsA			
Mean (s.d.)	0.42 (0.49)	0.50 (0.70)	0.45 (0.55)
Median (range)	0.38 (0.00, 2.00)	0.13 (0.00, 2.13)	0.25 (0.00, 2.13)
IQR	0.00, 0.63	0.00, 0.81	0.00, 0.63
0, *n* (%)	16 (25.0)	8 (34.8)	24 (27.6)
>0, *n* (%)	32 (50.0)	12 (52.2)	44 (50.6)
Missing, *n* (%)	16 (25.0)	3 (13.0)	19 (21.8)
Participants with non-zero score			
Mean (s.d.)	0.64 (0.47)	0.83 (0.74)	0.69 (0.55)
Median (range)	0.50 (0.13, 2.00)	0.56 (0.13, 2.13)	0.50 (0.13, 2.13)
IQR	0.38, 0.81	0.13, 1.50	0.31, 0.94
Change in HAQ-DI score between baseline and 24 months			
Mean (s.d.)	0.21 (0.33)	0.10 (0.25)	0.18 (0.31)
Median (range)	0.13 (–0.50, 0.88)	0.00 (–0.25, 0.63)	0.13 (–0.50, 0.88)
IQR	0.00, 0.50	0.00, 0.25	0.00, 0.38
Missing	16	5	21
Baseline HAQ-DI score	0	2	2
24-month HAQ-DI score	16	2	18
Both baseline and 24-month HAQ-DI score	0	1	1

HAQ-DI: HAQ Disability Index; IQR: interquartile range.

**Table 4.** Overall scaled HAQ-DI score (0–1) at baseline, and 12 and 24 months post-registration

	Enhanced surveillance (*N* = 64)	Standard care (*N* = 23)	Total (*N* = 87)
Baseline HAQ-DI scores			
All participants with a positive diagnosis of PsA			
Mean (s.d.)	0.09 (0.15)	0.11 (0.17)	0.10 (0.15)
Median (range)	0.00 (0.00, 0.71)	0.04 (0.00, 0.71)	0.00 (0.00, 0.71)
IQR	0.00, 0.13	0.00, 0.17	0.00, 0.13
Missing	0	3	3
0, *n* (%)	34 (53.1)	9 (39.1)	43 (49.4)
>0, *n* (%)	30 (46.9)	11 (47.8)	41 (47.1)
Missing, *n* (%)	0 (0.0)	3 (13.0)	3 (3.4)
Participants with non-zero score			
Mean (s.d.)	0.20 (0.16)	0.20 (0.19)	0.20 (0.17)
Median (range)	0.15 (0.04, 0.71)	0.13 (0.04, 0.71)	0.13 (0.04, 0.71)
IQR	0.08, 0.29	0.08, 0.25	0.08, 0.25
12 month HAQ-DI scores			
All participants with a positive diagnosis of PsA			
Mean (s.d.)	0.13 (0.15)	0.18 (0.22)	0.14 (0.17)
Median (range)	0.08 (0.00, 0.63)	0.08 (0.00, 0.67)	0.08 (0.00, 0.67)
IQR	0.00, 0.21	0.00, 0.33	0.00, 0.23
Missing	11	4	15
0, *n* (%)	18 (28.1)	7 (30.4)	25 (28.7)
>0, *n* (%)	35 (54.7)	12 (52.2)	47 (54.0)
Missing , *n* (%)	11 (17.2)	4 (17.4)	15 (17.2)
Participants with non-zero score			
Mean (s.d.)	0.20 (0.15)	0.29 (0.22)	0.22 (0.17)
Median (range)	0.17 (0.04, 0.63)	0.25 (0.04, 0.67)	0.17 (0.04, 0.67)
IQR	0.08, 0.29	0.08, 0.48	0.08, 0.29
24 month HAQ-DI scores			
All participants with a positive diagnosis of PsA			
Mean (s.d.)	0.14 (0.16)	0.17 (0.23)	0.15 (0.18)
Median (range)	0.13 (0.00, 0.67)	0.04 (0.00, 0.71)	0.08 (0.00, 0.71)
IQR	0.00, 0.21	0.00, 0.27	0.00, 0.21
Missing	16	3	19
0, *n* (%)	16 (25.0)	8 (34.8)	24 (27.6)
>0, *n* (%)	32 (50.0)	12 (52.2)	44 (50.6)
Missing, *n* (%)	16 (25.0)	3 (13.0)	19 (21.8)
Participants with non-zero score			
Mean (s.d.)	0.21 (0.16)	0.28 (0.25)	0.23 (0.18)
Median (range)	0.17 (0.04, 0.67)	0.19 (0.04, 0.71)	0.17 (0.04, 0.71)
IQR	0.13, 0.27	0.04, 0.50	0.10, 0.31
Change in HAQ-DI scores between baseline and 24 months			
Mean (s.d.)	0.07 (0.11)	0.03 (0.08)	0.06 (0.10)
Median (range)	0.04 (–0.17, 0.29)	0.00 (–0.08, 0.21)	0.04 (–0.17, 0.29)
IQR	0.00, 0.17	0.00, 0.08	0.00, 0.13
Missing	16	5	21
Missing baseline HAQ-DI score	0	2	2
Missing 24-month HAQ-DI score	16	2	18
Missing both baseline and 24-month HAQ-DI score	0	1	1

HAQ-DI: HAQ Disability Index; IQR: interquartile range.

After adjustment for all covariates in the final model, the odds of achieving a HAQ-DI score of 0 were 36% lower in the ES arm compared with the SC arm [OR 0.64 (95% CI 0.17, 2.38)], and therefore, the odds of achieving a more favourable outcome on the HAQ-DI were observed to be greater in the SC arm. However, there was insufficient evidence of a difference between the two treatment groups (*P* = 0.51). After adjusting for the same covariates, the odds of achieving a higher (non-zero) HAQ-DI score were 12% higher in the ES arm compared with the SC arm [OR 1.12 (95% CI 0.67, 1.86)], suggesting that physical function at 24 months was observed to be poorer in the ES arm. However, again there was insufficient evidence of a difference between the two treatment groups (*P* = 0.66) (Table 5). The conclusions were consistent for the complete case analysis population.

**Table 5.** Primary endpoint analysis: HAQ-DI score at 24 months post-registration (primary analysis population)

	Parameter	Baseline	24 months	OR (ES:SC)	95% CI	*P*-value
Zero-inflated part	*N* (%) with HAQ-DI = 0					
	ES	34 (53.1)	16 (25.0)	0.64	0.17, 2.38	0.51
	SC	9 (39.1)	8 (34.8)			
Non-zero part	Median (IQR)					
	ES	0.15 (0.08, 0.29)	0.17 (0.13, 0.27)	1.12	0.67, 1.86	0.66
	SC	0.13 (0.08, 0.25)	0.19 (0.04, 0.50)			

HAQ-DI: HAQ Disability Index; OR: odds ratio; IQR: interquartile range; ES: enhanced surveillance; SC: standard care.

A total of 50 (46 ES, 4 SC) participants completed a PsAID-12 questionnaire contemporaneously to at least one clinical assessment post-PsA diagnosis. Supplementary Fig. S2, available at *Rheumatology* online presents the participant profiles of PsAID-12 over time from date of PsA diagnosis, shows there is high variability in the scores between participants over time. The PASDAS score during at least one assessment post PsA diagnosis could be derived for a total of 21 (19 ES, 2 SC) participants. Supplementary Fig. S3, available at *Rheumatology* online on the participant profiles of the PASDAS score and corresponding components (Supplementary Fig. S4, available at *Rheumatology* online) from date of PsA diagnosis, show the sparseness of the data and variability in the scores between participants over time. Both the high variability over time in PsAID-12 and PASDAS and the small numbers especially in the SC arm precluded any meaningful statistical analysis.

No adverse events relating to trial procedures were reported during the trial. A total of 9 (0.4%) participants died post-registration, corresponding to 5 (0.4%) in the ES arm and 4 (0.4%) in the SC arm; there were no deaths in the primary analysis population.

## Discussion

The TUDOR trial set out to provide evidence to support implementation of a more proactive screening strategy to detect PsA at an earlier stage in a primary care setting than current standard practice. Although screening for PsA is advocated in some national guideline and audit tools [10, 11], it is rarely performed in routine practice and in no more that 50% of cases from dermatology clinics [29]. Previous studies that have suggested a delay in diagnosis may adversely affect outcome have been limited to patient cohorts already diagnosed in secondary care [6–8] and as such are likely to suffer from selection bias. The target population in the current study comprised patients known to have psoriasis according to their coded general practice record but not recorded as having PsA. Hence, TUDOR provided a unique opportunity of investigating the benefit of detecting PsA in a population where screening has been recommended.

Whilst the study recruited to target, the number of participants diagnosed with PsA was fewer than predicted. There are several reasons that may account for the lower numbers of confirmed cases of PsA. The participants may not be truly representative of a primary care population, although any such selection bias is more likely to favour healthcare-seeking participants with PsA coming forward for recruitment. In a primary care population, mild skin disease will occur more frequently than in secondary care cohorts and studies have shown that the prevalence of PsA increases with the severity of skin psoriasis [30, 31]. The trial was reliant on the participant’s GP referring any suspected case from enhanced surveillance on to secondary care through the usual care pathway. The onset of the COVID-19 pandemic occurred during the course of the trial and may well have deterred or delayed referral to secondary care. Clearly the pandemic had a major effect on hospital visits that were required in order to achieve confirmation of a PsA diagnosis, especially during the later course of the study, and as a consequence may have preferentially affected the standard care arm. It also took longer than expected for the hospital rheumatologist to commit to a diagnosis of PsA. Hence there may still be cases of undiagnosed PsA that have not been picked up in either arm of the study for any or a combination of the reasons stated. Alternatively, the amount of undiagnosed PsA may be close to what we have found and will need to be factored into future similar studies performed in primary care.

Notwithstanding the challenges and limitations faced in conducting our study there was insufficient evidence for a beneficial effect of enhanced surveillance compared with standard care in a primary care setting. There was less deterioration in physical function observed in the standard care arm than in enhanced surveillance, as measured by HAQ-DI as the primary outcome measure, although this was not statistically significant. The findings can be interpreted in several ways. Enhanced surveillance may detect cases of PsA that reflect inherently mild disease with better outcome than cases detected by routine care. This supposition is supported by finding a low mean baseline HAQ-DI in confirmed PsA cases in both arms (0.28 ES and 0.34 SC, respectively), compared with a mean baseline HAQ of 0.71 in patients presenting with PsA to secondary care in a Dublin study of early PsA [32]. Alternatively, the primary outcome we used may not have been sufficiently discriminatory to detect a difference, although the HAQ-DI has been used in other studies to show differences in outcome related to delayed diagnosis [7, 8]. It is possible that individual components of HAQ-DI such as hand function are more discriminatory. We did need to account for a sizeable proportion of participants having a zero score in HAQ at baseline that may also have diminished the ability to detect a difference. However, the other secondary outcomes used, namely PsAID-12 and PASDAS, showed considerable variability over time in those participants where data was available, making them less likely to be of value as a primary outcome. Finally, an endpoint of 24 months may be too soon to detect a benefit from enhanced surveillance, given that any beneficial effects of treatment such as early intervention with conventional disease-modifying agents followed by biologics may need to be modelled over a longer course of time.

There are several strengths to the TUDOR trial. Direct recruitment from primacy care allowed a wider, more representative population of patients with psoriasis to be investigated prospectively for the effectiveness of earlier detection of PsA. Participants in the SC arm were blinded to the main purpose of the trial, as were most GPs apart from the lead for the practice. Patients in both arms were referred to secondary care following the usual referral pathways. Once reaching secondary care, formal assessment was undertaken by trained clinicians. A limitation is not reaching the target number of patients receiving a final diagnosis of PsA, therefore the trial was underpowered for detecting the pre-specified treatment effect. There was also a large imbalance between the arms in the numbers of patients diagnosed with PsA, further reducing the power for the primary comparison. Such limitations could potentially be overcome by extending the period of follow-up, albeit that would now be unblinded. Despite recruiting from mixed populations, patients were mainly from white ethnic background, possibly reflecting the difficulty in offering translation to relevant languages in postal invitations.

An alternative interpretation of the findings is that consideration needs to be given to screening a population which is at more risk of developing severe forms of PsA rather than inviting all-comers from primary care with a record of having psoriasis. For instance, there is now well-established evidence that the presence of nail psoriasis [33], obesity [34], and severity of psoriasis [18] and/or comorbidities such as uveitis [35, 36] identify individuals with psoriasis at heightened risk of developing PsA. Hence, dermatology clinics are an additional target for applying a screening strategy. Pharmacists are also well positioned to select patients who attend for repeat prescriptions. Biomarkers are also likely to play an important role selecting more vulnerable populations. Whilst we can base no firm conclusions from the TUDOR trial, it potentially challenges the value of routine screening for PsA in a primary care psoriasis population with no additional risk factors for PsA.

## Supplementary Material

keae374_Supplementary_Data

## Data Availability

The data underlying this article are available in the article and in its online supplementary material.
