# Supplementary material for: Enhanced surveillance for the detection of psoriatic arthritis in a UK primary care psoriasis population: results from the TUDOR trial
Source: Rheumatology (Oxford). 2024 Jul 22;64(4):1750–9. doi: 10.1093/rheumatology/keae374 (PMC11962868; doi:10.1093/rheumatology/keae374)
Supplement: keae374_Supplementary_Data [file keae374_supplementary_data.docx]

**Supplementary information**

Supplementary Table 1: Referral time point for those participants diagnosed with PsA

|  | **Enhanced Surveillance Arm (N = 64)** | **Standard of Care Arm (N = 23)** | **Total (N = 87)** |
| --- | --- | --- | --- |
|  |  |  |  |
| Baseline visit referral | 43 (67.2%) | 0 (0.0%) | 43 (49.4%) |
| 12 month visit referral | 9 (14.1%) | 0 (0.0%) | 9 (10.3%) |
| 24 month visit referral | 4 (6.3%) | 15 (65.2%) | 19 (21.8%) |
| Ad-hoc referral | 8 (12.5%) | 8 (34.8%) | 16 (18.4%) |
| --- Ad-hoc Baseline referral | 0 (0.0%) | 1 (4.4%) | 1 (1.2%) |
| --- Ad-hoc referral within 12 months of registration | 5 (7.8%) | 7 (30.4%) | 12 (13.8%) |
| --- Ad-hoc referral between 12 & 24 months post registration | 3 (4.7%) | 0 (0.0%) | 3 (3.5%) |

Supplementary Figure 1. Histograms of the scaled overall HAQ-DI Score at baseline, 12 and 24 months by treatment group


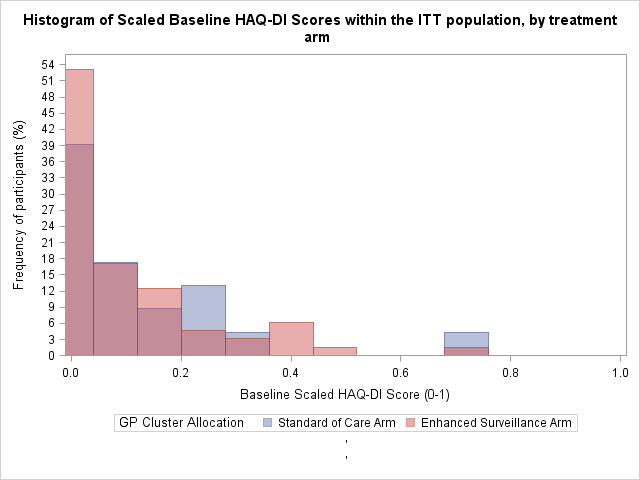

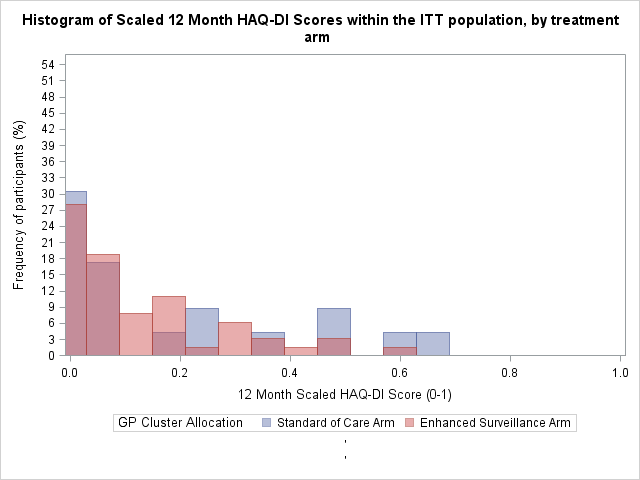

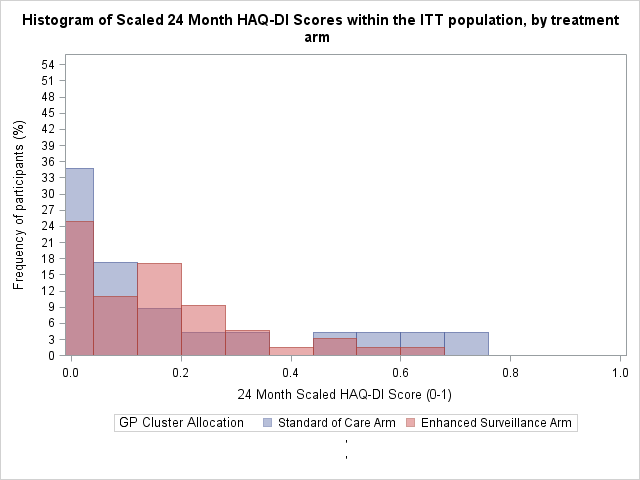

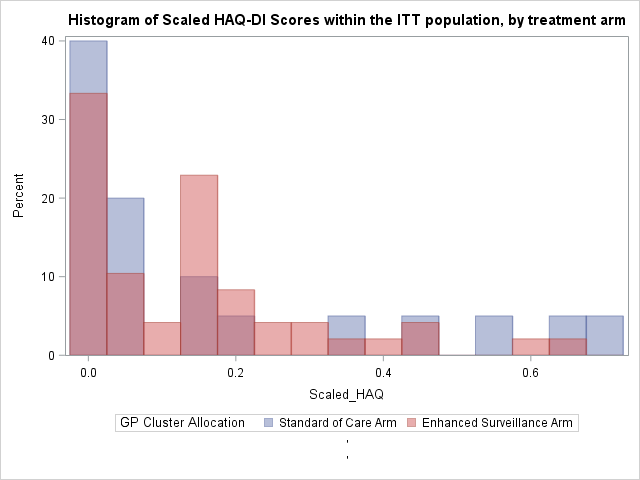

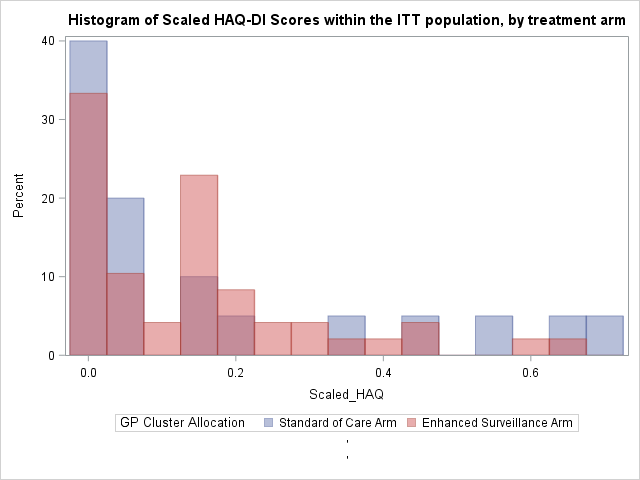


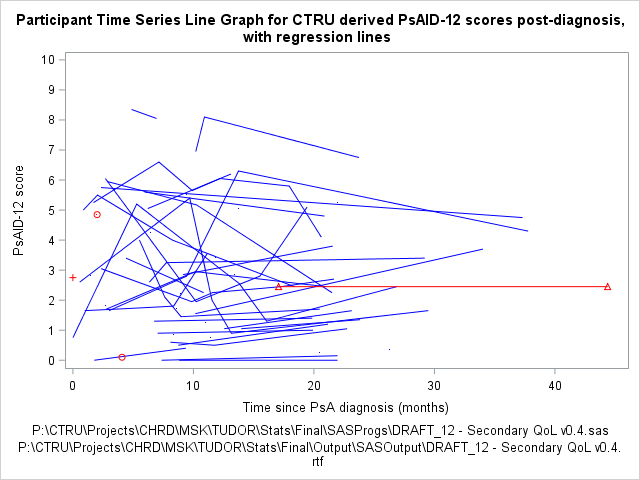


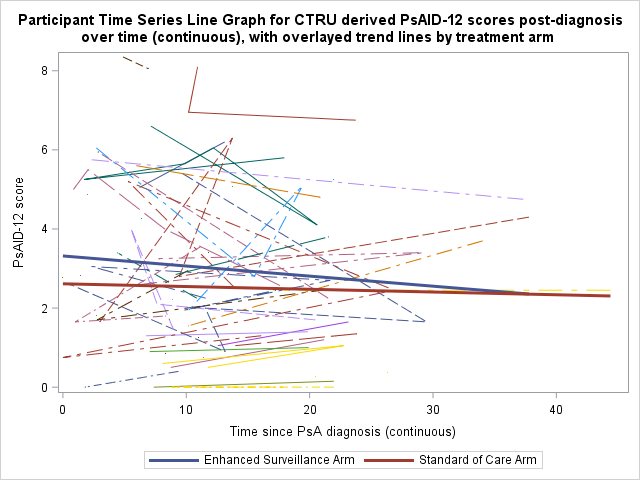


Supplementary Figure 2. Participant profiles of PsAID-12 over time since PsA diagnosis by treatment group

Supplementary Figure 3. Participant profiles of PASDAS score over time since PsA diagnosis by treatment group


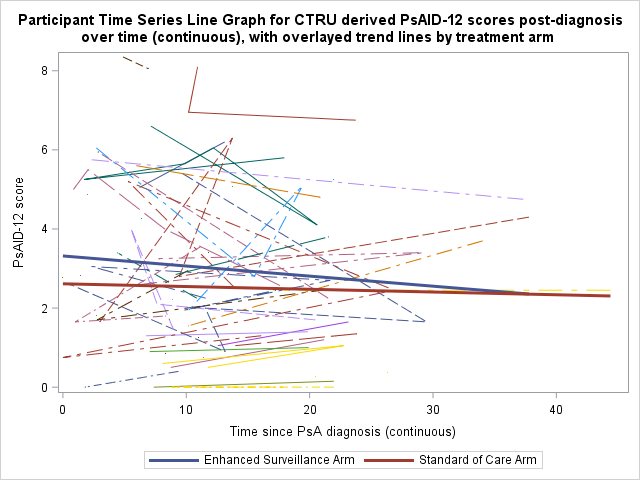

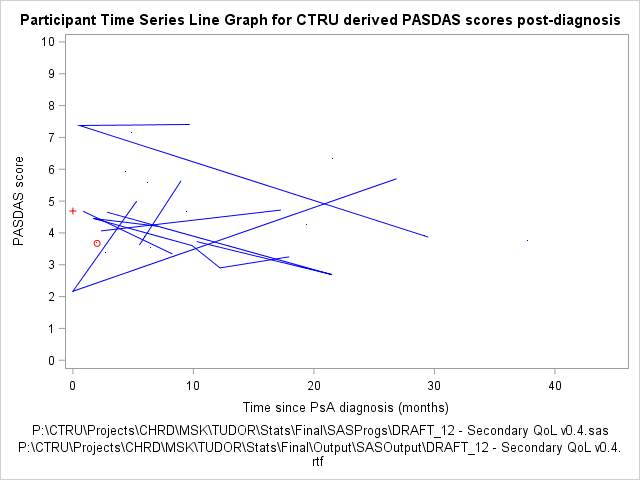


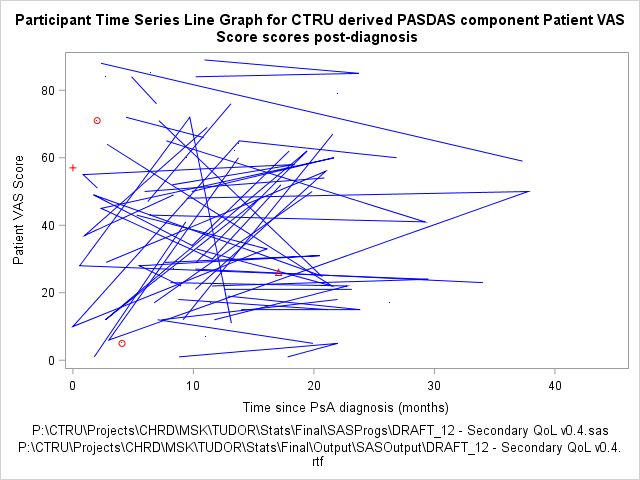
**
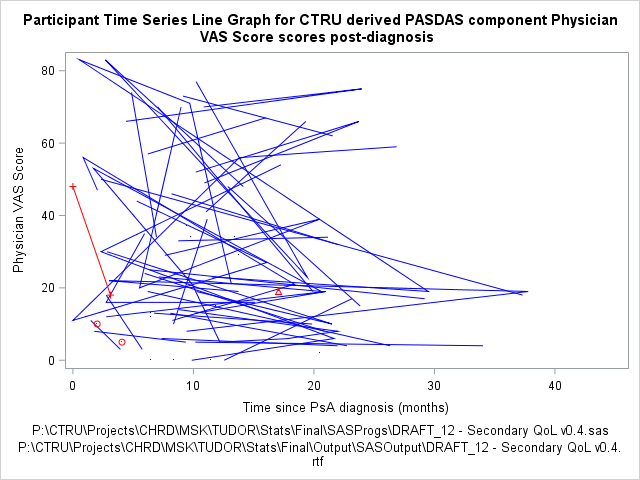
**

**
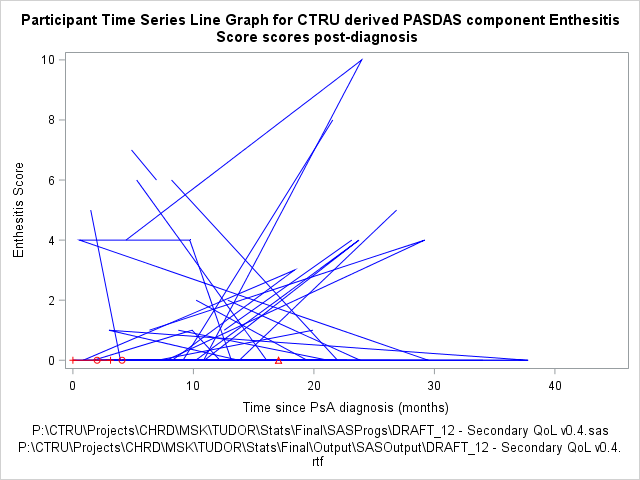

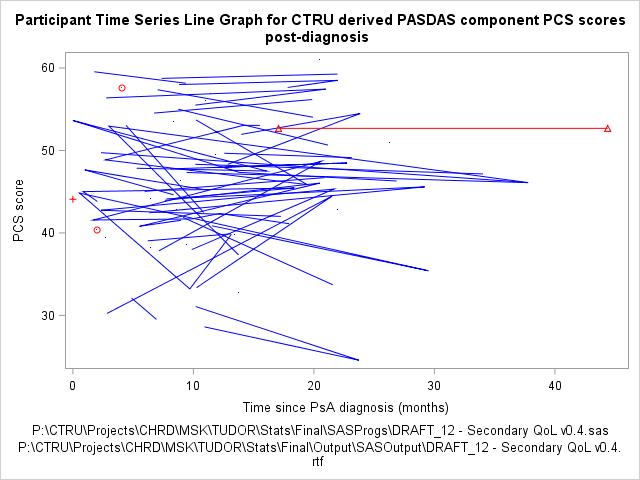
**

**
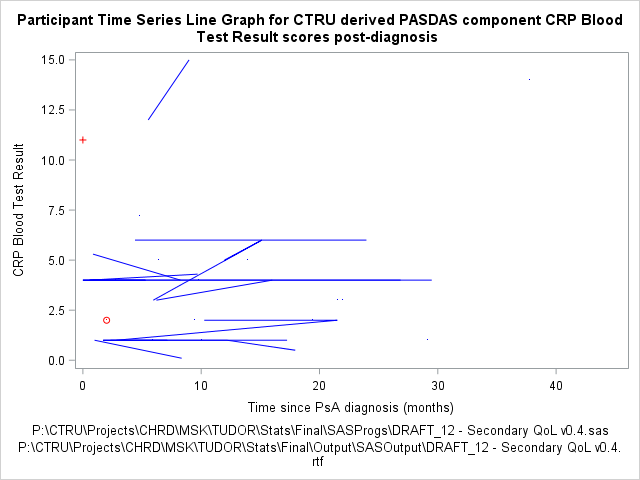

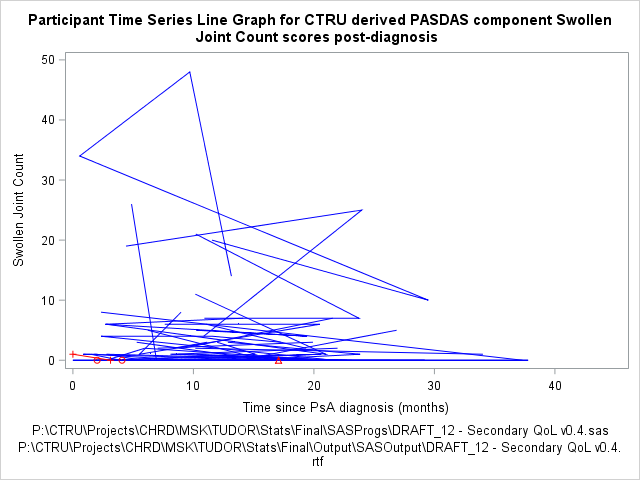

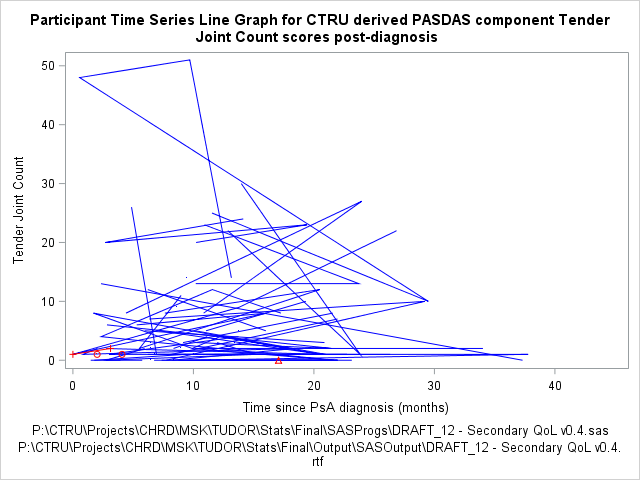
**

***
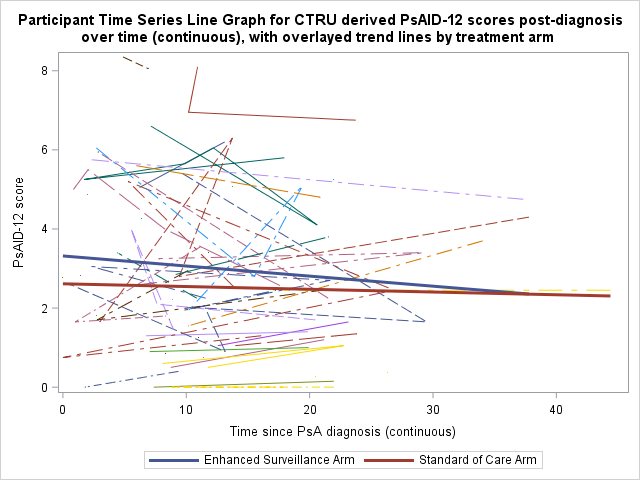
*
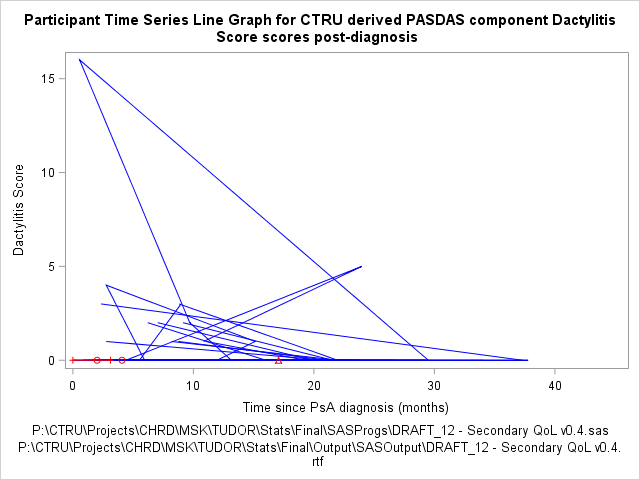
**

Supplementary Figure 4. PASDAS component scores over time since PsA diagnosis by treatment group
